# Temporary Global Amnesia With Insular Infarction in a Young Female: A Case Report

**DOI:** 10.7759/cureus.76534

**Published:** 2024-12-28

**Authors:** Tetsuya Akaishi, Yoko Suzuki, Yuichi Kawabata, Yusho Ishii, So Itoi, Hiroko Sato, Yutaro Sugiyama, Akimitsu Nishiyama

**Affiliations:** 1 Department of Education and Support for Regional Medicine, Tohoku University Hospital, Sendai, JPN; 2 Division of Internal Medicine, Izumi Himawari Clinic, Sendai, JPN; 3 Department of Stroke Neurology, Kohnan Hospital, Sendai, JPN; 4 Department of Rheumatology, Tohoku University, Sendai, JPN; 5 Department of Cardiology, Sendai Kosei Hospital, Sendai, JPN; 6 Division of Internal Medicine, Miyagi Central Hospital, Sendai, JPN

**Keywords:** antiphospholipid syndrome, extralimbic system, global amnesia, insular infarction, systemic lupus erythematosus

## Abstract

Several neurological conditions, including transient global amnesia (TGA), may present an isolated sudden-onset temporary amnestic symptom. TGA is a benign, self-remitting neurological condition associated with hippocampal dysfunction. Meanwhile, certain other neurological conditions, such as cerebral ischemic stroke and hippocampal epilepsy, require appropriate therapeutic interventions. An isolated temporary amnestic symptom caused by extra-limbic brain regions has not been widely reported yet.

We report a case of a 37-year-old female with systemic lupus erythematosus (SLE) who suddenly developed a transient amnestic symptom, with no other neurological symptoms, during an outdoor activity on a hot sunny day. Anterograde amnesia resolved after approximately two hours from the onset. She visited the hospital three days later. Brain MRI revealed an acute infarction in the left anterior insular cortex. A 2 mm thin-slice MRI on both hippocampi revealed no diffusion restriction. She was diagnosed with insular cortex infarction, possibly associated with SLE. Single antiplatelet therapy with aspirin 100 mg/day was started on the same day and oral prednisolone with 10 mg/day was started on the next day.

This report is the first of its kind to describe a possible association between temporary amnesia and insular cortex damage. This case implies that the anterior insular cortex may play a potential role in forming episodic memories. Damages in this area may present a sudden-onset transient amnestic symptom. Clinicians should pay close attention to this non-hippocampal area on brain MRI when investigating amnestic patients.

## Introduction

One of the most common conditions presenting an isolated sudden-onset temporary amnestic symptom is transient global amnesia (TGA) [1,2]. TGA is a benign functional neurological condition, often accompanied by self-remitting unilateral or bilateral punctate hippocampal diffusion restrictions [3-5]. This benign neurological condition is fairly known, but its diagnosis must be carefully made by excluding other similar non-benign neurological conditions like hippocampal epilepsy and hippocampal infarction. Brain MRI imaging and electroencephalogram (EEG) are necessary steps to establish a correct diagnosis [6,7]. Currently, irrespective of whether benign or non-benign, temporary global amnesia is considered to primarily derive from disturbances in the components of the Papez circuit, including the hippocampus, thalamus, and cingulate gyrus [8-11]. We discuss a case of a young female who presented with a sudden-onset temporary global amnesia, potentially caused by unilateral insular cortex infarction associated with systemic lupus erythematosus (SLE). This is the first case report to describe a possible association between sudden-onset temporary global amnesia and insular infarction.

## Case presentation

A 37-year-old left-handed female visited an outdoor facility in Japan with her family on a hot sunny day with the highest temperature of 27.9 ℃. She drove the car herself to the facility and arrived there at approximately 11:00 AM. At approximately 12:30 PM, when she was standing in line to buy an ice cream, she suddenly started to repeatedly ask “Where are we?” and “How did I reach this place today?” Until 02:30 PM, she continued to ask her family members the aforementioned questions every 5-10 minutes. After 02:30 PM, her anterograde amnesia resolved, and she stopped asking the questions. For a while, she had no memory from the morning before driving to the place till 02:30 PM. She gradually recovered her lost memories caused by retrograde amnesia from the morning to the onset at 12:30 PM, but the lost memories caused by anterograde amnesia between 12:30 PM and 02:30 PM were not recovered. She did not visit the hospital until she was advised to do so by an acquaintance of hers three days after the amnestic episode.

The patient had a past medical history of chronic headaches, and she had recently been diagnosed with systemic lupus erythematosus (SLE). The first clinical episode associated with SLE had been polyarticular pain in her hands, which had started eight months before the amnestic episode. She had no history of oral medication or cigarette smoking. Her blood test results two weeks before the amnestic episode are shown in Table 1. Based on the subsequent amnestic episode, possible coexistence of antiphospholipid syndrome (APS) was also suspected and additional blood test parameters related to the disease were further evaluated. The results of the APS-associated blood test results are summarized in the upper half of Table 2. Based on these findings, possible diagnoses included central nervous system (CNS) lupus and SLE-associated cerebral infarction, such as SLE with APS.

**Table 1:** Blood test results two weeks before the amnestic episode This data was obtained two weeks before the amnestic episode. No treatments for SLE had not been started at this point ANA: anti-nuclear antibodies; CH50: 50% hemolytic complement; CRP: C-reactive protein; MMP-3: matrix metalloproteinase-3; SLE: systemic lupus erythematosus

Variables	Result	Reference range
ANA (homogeneous pattern)	1:1,280	≤1:40
ANA (speckled pattern)	1:40	≤1:40
ANA (nucleolar pattern)	1:40	≤1:40
MMP-3	20 ng/ml	17.3–59.7
Anti-SSA antibody	>240 U/ml	≤7.0
Anti-SSB antibody	3.9 U/ml	≤7.0
Anti-ds-DNA IgG	109.8 IU/ml	≤10.0
Rheumatoid factor	8 IU/ml	≤15
C3	90 mg/dl	80–140
C4	10.2 mg/dl	11.0–34.0
CH50	13 U/ml	30–45
Total protein	8.0 g/dl	6.5–8.2
Albumin	4.3 g/dl	3.8–5.2
White blood cell count	5,680/μl	3,500–9,700
Neutrophils	81.8%	42.0–74.0
Lymphocytes	13.6%	18.0–50.0
Hemoglobin	12.1 g/dl	F: 11.2–15.2
Platelet count	237,000/μl	140,000–379,000
CRP	1.63 mg/dl	≤0.30

**Table 2:** Post-amnestic blood test data related to antiphospholipid syndrome before and after starting SLE treatments ANA: anti-nuclear antibodies; β2GP1: beta-2-glycoprotein 1; SLE: systemic lupus erythematosus

Variables	Result	Reference range
Three days after the amnestic episode (before starting SLE treatments)		
ANA (homogeneous pattern)	1:1,280	≤1:40
ANA (speckled pattern)	1:40	≤1:40
ANA (nucleolar pattern)	1:640	≤1:40
Anti-cardiolipin IgG	10.7 U/ml	≤20.0
Anti-cardiolipin IgM	36.5 U/ml	≤20.0
Anti-β2GP1 IgG	35.9 U/ml	≤20.0
Anti-β2GP1 IgM	29.4 U/ml	≤20.0
D-dimer	1.7 μg/ml	≤1.0
Three months after the amnestic episode (after starting SLE treatments)		
Anti-cardiolipin IgG	3.4 U/ml	≤20.0
Anti-cardiolipin IgM	12.5 U/ml	≤20.0
Anti-β2GP1 IgG	7.7 U/ml	≤20.0
Anti-β2GP1 IgM	7.4 U/ml	≤20.0

**Figure 1:**
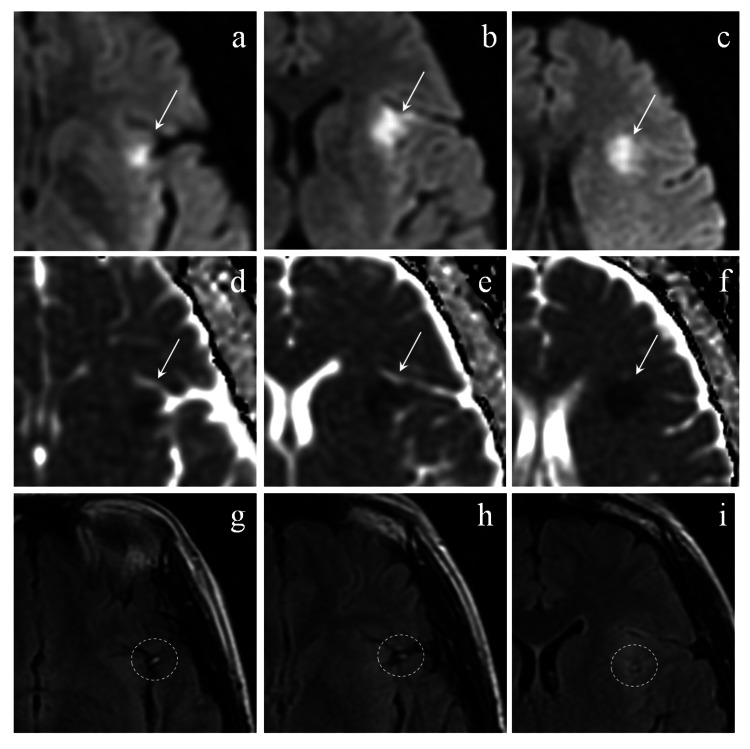
Initial MRI imaging with 6 mm-thickness slices three days after amnestic episode (a-c) Axial diffusion-weighted imaging with the parameters of repetition time (TR)/echo time (TE) = 5,000/84.3 ms. White arrows show hyperintensities in the left anterior insular cortex. (d-f) Axial apparent diffusion coefficient map with the parameters of TR/TE = 5,000/84.3 ms. White arrows show signal loss at the same site of diffusion restriction area. (g-i) Axial fluid-attenuated inversion recovery with TR/TE = 10,000/139 ms. White circles show hyperintense MCA signs in the left Sylvian fissure. MCA: middle cerebral artery; MRI: magnetic resonance imaging

**Figure 2:**
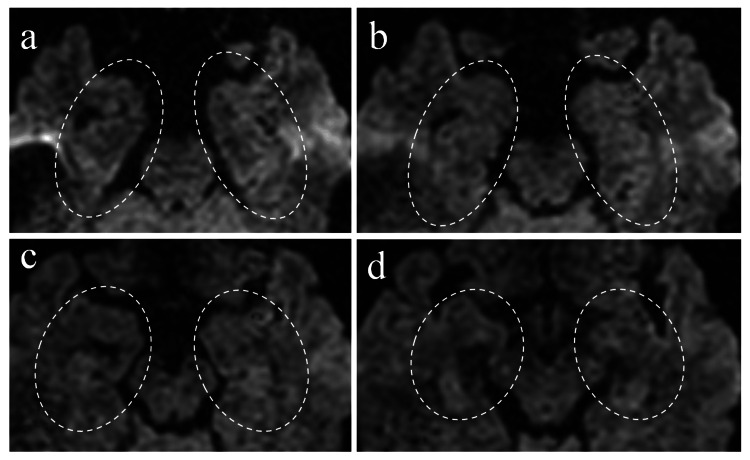
MRI imaging with 2 mm-thickness thin-slice three days after amnestic episode showing the absence of hippocampal lesions No diffusion restriction area was detected in the hippocampal areas (white ellipses) even with the careful investigation utilizing a thin-slice MRI three days after the amnestic episode, making the diagnosis of TGA less likely. Axial diffusion-weighted imaging with TR/TE = 5000/84.3 ms MRI: magnetic resonance imaging; TGA: transient global amnesia

The patient's brain MRI images three days after the amnestic episode are shown in Figure 1. Diffusion restrictions were observed in the left insular cortex, with an intraarterial hyperintense signal on FLAIR images inside the middle cerebral artery (MCA) in the Sylvian fissure. Hippocampal areas were carefully investigated with 2 mm thin-slice MRI imaging, but there were no detectable ischemic hippocampal lesions (Figure 2). These findings supported the diagnosis of SLE-associated cerebral infarction rather than CNS lupus.

**Figure 3:**
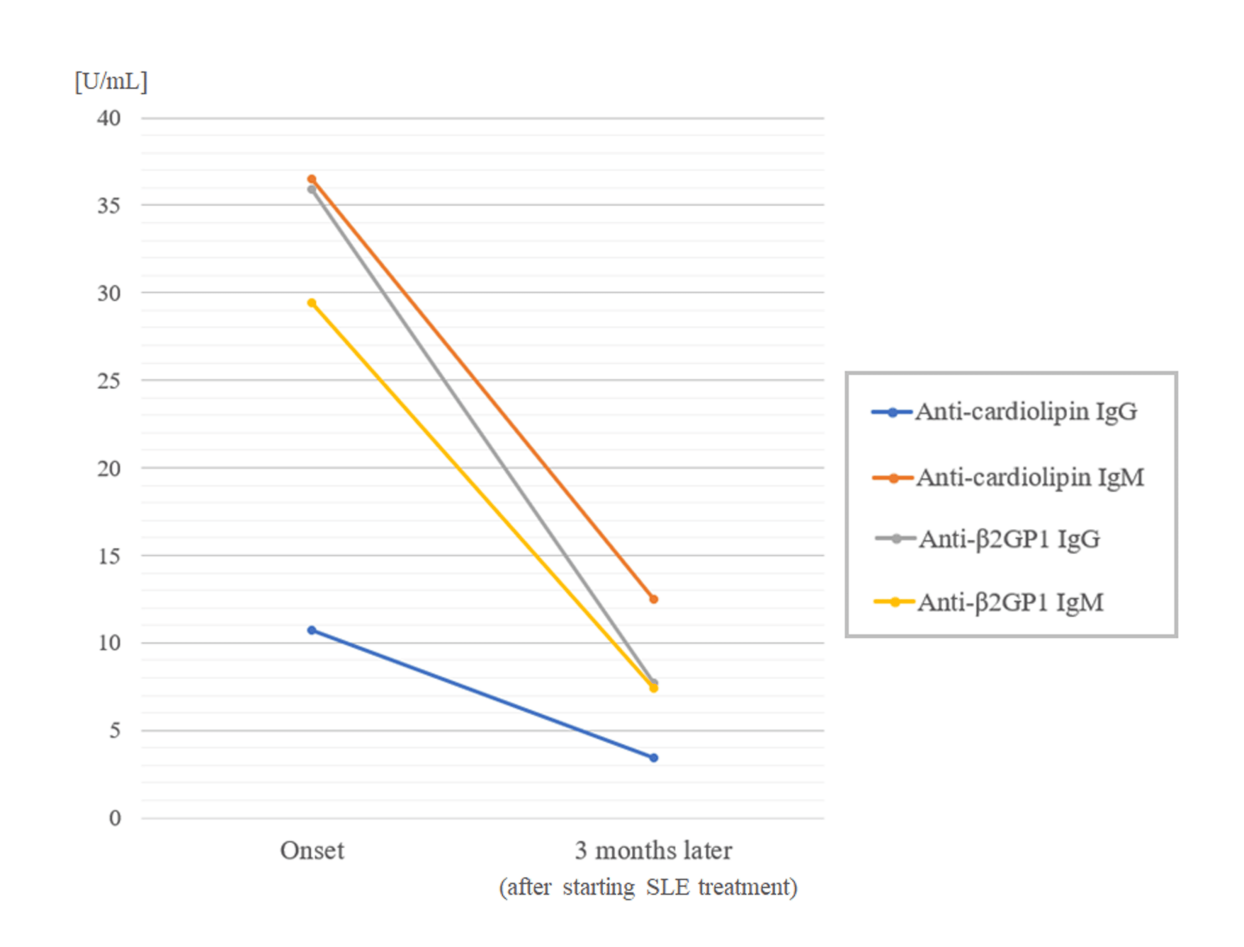
Changes in the serum autoantibody titers related to antiphospholipid syndrome All of the four evaluated titers related to antiphospholipid syndrome decreased after starting oral prednisolone β2GP1: beta-2-glycoprotein 1; SLE: systemic lupus erythematosus

Based on these findings, single antiplatelet therapy with aspirin 100 mg/day was started on the same day and oral prednisolone with 10 mg/day was started on the next day. About one month later, warfarin (4 mg daily) was additionally started since possible coexistence of APS was suspected. The antiphospholipid antibody titers three months after the neurological onset and the start of SLE treatment are shown in the lower half of Table 2. Each of the four APS-related antibodies showed a dramatic decrease in its titer after the SLE treatment (Figure 3). Currently, after six months of the neurological episode, the patient is free of any symptoms or neurological sequelae including short-term memory.

## Discussion

We delve into the case of a young female who developed sudden-onset transient amnesia caused by an anterior insular cortex infarction possibly associated with the comorbid SLE. A possible diagnosis of TGA caused by undetectable hippocampal involvement accompanied by insular cortex involvement was initially considered, but it was unlikely because the insular cortex and hippocampus are supplied by different major cerebral arteries. The insular cortex is supplied by MCA, while the hippocampus is supplied by the posterior cerebral artery. Moreover, there was no diffusion restriction on either side of the hippocampi, even by utilizing 2 mm thin-slice MRI imaging three days after the neurological onset. Therefore, the most reasonable diagnosis in this case was a sudden-onset transient amnestic episode caused by left insular cortex infarction. The cause of insular cortex infarction was APS accompanied by undiagnosed SLE.

The insular cortex constitutes the surface area of the insular lobe, which is covered by frontal and temporal lobes. Although the exact function of the insular cortex remains unknown, it is suggested to play a role in multisensory attention, visual awareness, multimodal sensory integration, and emotional functions [12-14]. A recent literature review involving 49 patients with insular stroke found that atypical presentations include hemispatial awareness deficits, but amnestic symptoms were not mentioned [15]. Strong structural and functional connections between the anterior insula and anterior cingulate cortex, a part of the limbic system, have been reported [16-18]. Therefore, amnestic symptoms with anterior insular infarction based on disturbed attention-related alertness may be theoretically possible. Further case reports are needed to gain deeper insights into the possible manifestation of isolated global amnesia with insular infarction.

As can be inferred from the present report, it is very dangerous to carelessly make a diagnosis of TGA based only on the clinical manifestation of sudden-onset temporary amnestic symptoms without performing brain MRI at adequate timings. Empirically, many undiagnosed non-benign amnestic cases do not visit hospitals or are carelessly diagnosed with TGA by the first-contact physician without necessary diagnostic approaches. One of the causative factors underlying this problem would be that the currently prevailing disease name of TGA purely comprises three clinical characteristics of transience, globality, and amnesticity, although many non-benign neurological conditions fulfill these criteria.

Unfortunately, many physicians carelessly assume that all patients with sudden-onset temporary global amnesia have TGA and they do not require any diagnostic examinations or treatments. Clinicians must keep in mind that patients with sudden-onset temporary amnestic symptoms, even without other accompanying neurological deficits, must be swiftly investigated with MRI. To detect small ischemic diffusion restriction areas, thin-slice MRI will help with the detection of small spotty diffusion restriction areas, whether these are benign or non-benign. In some cases, a follow-up MRI seven days from the amnestic episode will be needed to correctly discriminate ischemic infarction from TGA [19]. A way to avoid such careless misdiagnosis is to apply new criteria like “benign TGA” and “non-benign TGA”, with the former corresponding to conventional TGA. By using such terms, physicians can intuitively understand that not all cases with temporary global amnesia are benign. Discussions will be needed to seek and devise better disease criteria for patients with isolated sudden amnestic symptoms.

## Conclusions

Our findings imply that the anterior insular cortex is closely associated with the formation of episodic memory. Insular infarction may present transient amnestic symptoms resembling TGA. Further case reports are needed to establish the relationship between the insular lobe and episodic memory formation. All cases with an isolated sudden-onset transient amnestic symptom must be carefully studied with brain MRI so as not to overlook brain infarctions both in hippocampal and non-hippocampal areas. Thin-slice MRI of the pertinent brain area is useful for detecting small infarctions. Persisting hyperintensities on diffusion-weighted MRI over one week suggest ischemic neurological conditions requiring appropriate diagnostic and therapeutic approaches. Clinicians should be mindful that TGA is a diagnosis of exclusion, and the diagnosis can only be made after careful tests including repeated MRIs over one week from the amnestic onset as needed.
